# Inhibitory Effect of* Loranthus parasiticus* on IgE-Mediated Allergic Responses in RBL-2H3 Cells

**DOI:** 10.1155/2016/8742562

**Published:** 2016-09-27

**Authors:** Jae-Myung Yoo, Ju-Hye Yang, Young Soo Kim, Won-Kyung Cho, Jin Yeul Ma

**Affiliations:** Korean Medicine (KM) Application Center, Korea Institute of Oriental Medicine (KIOM), Daegu 41062, Republic of Korea

## Abstract

The mistletoe* Loranthus parasiticus *has been used as a compound for traditional medicine in Northeast Asia for a long time and is known to possess neuroprotective action. Nonetheless, the effect of* Loranthus parasiticus* on allergic responses remains unknown. In the present study, we evaluated whether the water extract of* Loranthus parasiticus* (LPE) could inhibit IgE-mediated allergic responses in RBL-2H3 cells. LPE inhibited the release of *β*-hexosaminidase (IC_50_, 184.5 *μ*g/mL) and the formation of tumor necrosis factor-*α* (IC_50_, 84.27 *μ*g/mL), interleukin-4 (IC_50_, 93.43 *μ*g/mL), prostaglandin E_2_ (IC_50_, 84.10 *μ*g/mL), prostaglandin D_2_, and leukotriene C_4_ (IC_50_, 43.27 *μ*g/mL) in a concentration-dependent manner. Moreover, LPE inhibited phosphorylation of Syk, PLC*γ*1/2, PKC*δ*, ERK, JNK, p38, and Akt. In the late phase, LPE decreased 5-lipoxygenase phosphorylation and COX-2 expression but not cPLA_2_ phosphorylation. Additionally, LPE included total phenolic compounds (10.72 mg/g dry weight) and total flavonoids (56.20 mg/g dry weight). These results suggest that the phenolic compounds or flavonoids contained in LPE may be associated with antiallergic activity. The phenolic compounds and flavonoids in LPE are antiallergic phytochemicals capable of inhibiting the activation of the Fc*ε*RI signaling cascade in mast cells. Such effects may provide further information for the development of a phytomedicine for allergic diseases.

## 1. Introduction

Mistletoe, a semiparasitic plant, is widely distributed across the globe and has been used as a constituent of traditional medicine in Northeast Asia for centuries [1]. Five species of mistletoe are widely distributed in the Republic of Korea.* Viscum album *L., known as European mistletoe, and* Loranthus parasiticus*, known as Mulberry mistletoe, are mainly used for traditional medicine in the Republic of Korea [2]. Mistletoe possesses various beneficial effects, such as anticancer, antiobesity, neuroprotection, antioxidant, and anti-inflammation activities [3]. The extract of* Viscum album *L., known as Iscador, has been used in anticancer therapy in Europe because it possesses strong anticancer action [4]. Such effects of mistletoe are associated with various bioactive compounds, including lectins, viscotoxins, triterpenes, sesquiterpene lactones, flavonoids, and phenolic compounds [5]. Nonetheless, the biological effect of* Loranthus parasiticus* is still unknown with the exception of its neuroprotective effects [6].

Anaphylactic shock is a type I allergy that is closely associated with acute inflammation [7]. The inflammatory response is associated with the degranulation of immunoglobulin E- (IgE-) sensitized mast cells or basophilic cells [8]. These cells express Fc*ε*RI receptors known as the high-affinity IgE receptor located on the plasma membrane [8]. When IgE-sensitized mast cells are stimulated by antigens, the cells liberate various inflammatory mediators, including tumor necrosis factor-*α* (TNF-*α*), interleukin-4 (IL-4), prostaglandin E_2_ (PGE_2_), prostaglandin D_2_ (PGD_2_), and leukotriene C_4_ (LTC_4_) with *β*-hexosaminidase, a biomarker of degranulation, through activation of the Fc*ε*RI signaling cascade [8–10]. Moreover, PGD_2_ and LTC_4_ are involved in chronic inflammation in asthma or allergic rhinitis [11, 12]. Therefore, anaphylactic shock is very important clinically.

In this study, we found that the extract of* Loranthus parasiticus* (LPE) possessed antiallergic activity in IgE-mediated allergic responses in mast cells and demonstrate how LPE inhibits allergic responses in the above cells. In conclusion, the results may provide further information for the development of a phytomedicine for allergic diseases.

## 2. Materials and Methods

### 2.1. Reagents

MEM-*α* medium, 1x DPBS, fetal bovine serum (FBS), penicillin, and streptomycin were purchased from GE Healthcare Life Sciences (Hyclone*™*, Logan, UT, USA). The EZ-Cytox cell viability assay kit was obtained from DAEILLAB SERVICE Co. (Seoul, Korea). Specific antibodies against phospho-protein kinase B (Akt; #9271), phospho-cytosolic phospholipase A_2_ (cPLA_2_; #2831), phospho-extracellular signal-regulated kinase 1/2 (ERK; #9101), phospho-c-Jun* N*-terminal kinase 1/2 (JNK; #9251), phospho-Src family protein kinase (Lyn; #2731), phospho-p38 (#9211), phospho-protein kinase C*δ* (PKC*δ*; #2055), phospho-phospholipase C*γ*1/2 (PLC*γ*1/2; #2821, #3871, resp.), and phospho-spleen tyrosine kinase (Syk; #2710) and cyclooxygenase-2 (COX-2; #4842) were obtained from Cell Signaling Technology, Inc. (Beverly, MA, USA). Specific antibodies against phospho-feline yes-related protein (Fyn; orb128087) and *β*-actin (sc-47778) were obtained from Biorbyt Ltd. (Cambridge, UK) and Santa Cruz Biotechnology, Inc. (Dallas, TX, USA), respectively. A specific antibody against 5-lipoxygenase (5-LO; 10007820) and EIA kits for LTC_4_, PGD_2_, and PGE_2_ were obtained from Cayman Chemical Co. (Ann Arbor, MI, USA). ELISA kits for IL-4 and TNF-*α* were purchased from e-Bioscience, Inc. (Science Center Drive, San Diego, USA). Dinitrophenyl-human serum albumin (DNP-HSA), DNP-IgE, Folin-Ciocalteu reagent, caffeic acid, diethylene glycol, quercetin, and 4-nitrophenyl* N*-acetyl-*β*-*D*-glucosaminide (p-NAG) were purchased from Sigma-Aldrich Co. (St. Louis, MO, USA). All other chemicals for this study were of analytical grade.

### 2.2. Preparation of* Loranthus parasiticus *Extract

LPE was prepared according to a modification of a process reported previously [13];* Loranthus parasiticus* was obtained from the Yeongcheon Oriental Herbal Market (Yeongcheon, Korea) and then identified by Dr. Ki-Hwan Bae, a Professor Emeritus at the College of Pharmacy, Chungnam National University (Daejeon, Korea).* Loranthus parasiticus* (1 kg) was boiled in distilled water (10 liter) for approximately 3 h at 115°C. The aqueous extract was filtered through a testing sieve (Aperture 500 *μ*m and 150 *μ*m). The filtered extract was filtered through a 60 *μ*m nylon net filter (Millipore, MA, USA) and deposited overnight. The supernatant was lyophilized, and then the dried pellet was stored at −20°C until use. The powder of LPE was dissolved in 10% dimethyl sulfoxide (DMSO) solution for all experiments.

### 2.3. Determination of Total Phenolic and Flavonoid Compounds

The amounts of total phenolic compounds and flavonoids in LPE were evaluated following previously reported methods [14]. LPE powder was dissolved using 20 mM PBS buffer (pH 7.4) to a final concentration of 100 mg/mL. The solution (0.33 mL) was mixed with 2.5 mL of distilled water and then incubated with 0.16 mL of Folin-Ciocalteu reagent for 5 min. The above solution was further incubated for 30 min in darkness after treatment with 10% sodium bicarbonate solution (0.3 mL). The absorbance at 760 nm was measured using a microplate reader (SpectraMax i3, Molecular devices, CA, USA). A standard curve was prepared to express the results as caffeic acid equivalents. Separately, to determine amounts of total flavonoids in LPE, 0.4 mL of LPE was added to 4 mL 90% diethylene glycol containing 0.4 mL of 1 N NaOH, and then the mixture was incubated for 1 h. The absorbance of the solution at 420 nm was measured using a microplate reader. A standard curve was prepared to express the results as quercetin equivalents.

### 2.4. Cell Culture

RBL-2H3 cells, a mast cell line originating from rat basophilic leukemia [15], were cultured in MEM-*α* medium including 10% FBS and antibiotics (100 U/mL penicillin and 100 *µ*g/mL streptomycin) at 37°C in a humidified atmosphere of 5% CO_2_. All the experiments contain a control group as a vehicle control group containing 0.1% DMSO.

### 2.5. Cell Viability Assay

Cell viability was evaluated by measuring the mitochondrial-dependent conversion from WST-1 to water-soluble tetrazolium salt [16]. In brief, RBL-2H3 cells were seeded on a 96-well plate (1 × 10^4^ cells/well) in MEM-*α* medium containing 10% FBS at 37°C overnight. The above cells were washed with 1x DPBS and then incubated with 50 ng/mL DNP-IgE. After 24 h, IgE-sensitized cells were preincubated with LPE (0 to 400 *μ*g/mL) in MEM-*α* medium with 1% FBS for 1 h, simultaneously mixed with 0.1 *μ*g/mL DNP-HSA and 10 *μ*L EZ-Cytox reagent, and then further incubated for 4 h. The cell viability of the above cells was determined by a microplate reader (450 nm).

### 2.6. *β*-Hexosaminidase Activity Assay

*β*-Hexosaminidase activity assay was performed following the previously reported method [17]. Supernatant (25 *μ*L) was added to 50 *μ*L p-NAG (10 mM) in 0.1 M sodium citrate buffer (pH 4.5) and then incubated for 1 h at 37°C. The reaction was finished by 0.1 M sodium carbonate buffer (pH 10.0). The absorbance was measured at 405 nm using a microplate reader.

### 2.7. Enzyme-Linked Immunosorbent Assay for IL-4 and TNF-*α*

To determine the amounts of TNF-*α* or IL-4 in cultured media, IgE-sensitized cells were preincubated with LPE in MEM-*α* medium with 1% FBS for 1 h and then stimulated with DNP-HSA for 4 h. All cultured media were centrifuged (17,000 ×g) for 10 min at 4°C, and then the samples were stored at −80°C until use. IL-4 and TNF-*α* were evaluated by ELISA kits according to the manufacturer's instruction.

### 2.8. Enzyme Immunoassay Analysis for LTC_4_, PGD_2_, and PGE_2_

To measure the levels of PGD_2_, PGE_2_, or LTC_4_ in cultured media, all samples were centrifuged and stored at −80°C until use. LTC_4_, PGD_2_, and PGE_2_ and were measured by EIA kits according to the manufacturer's instruction.

### 2.9. Immunoblotting Analysis

Immunoblotting analysis was determined using the previous method [17]. Blotted membranes were visualized using the ECL plus kit as a chemiluminescent reagent (Bio-Rad, Hercules, CA, USA) with an Imaging system (ChemiDoc Touch Imaging System, Bio-Rad, Hercules, CA, USA). The density levels of target proteins identified by a protein standard size marker (BIOFACT, Daejeon, Korea) were compared to those of a loading control (*β*-actin). The density of target protein bands was measured using ImageJ software (version 1.49v for Windows, NIH, USA).

### 2.10. Statistical Analyses

All experimental results were reported as means ± SD. One-way analysis of variance (ANOVA) was used for multiple comparisons (GraphPad Prism version 5.03 for Windows, San Diego, CA, USA). If there was a significant variation between treated groups, the Dunnett test was applied. Differences at the ^*∗*^ *P* < 0.05 and ^*∗∗*^ *P* < 0.01 levels were considered statistically significant.

## 3. Results

### 3.1. Profiles of Total Phenolic Compounds and Flavonoids in LPE

First, we investigated whether LPE includes phenolic compounds and flavonoids because these compounds from various mistletoes are known to possess various beneficial effects, such as antioxidant, neuroprotection, and anticancer effects [3]. LPE contained total phenolic compounds (10.72 ± 0.06 mg/g dry weight, the mean ± SD values of triple determinations) and total flavonoids (56.20 ± 0.40 mg/g dry weight, the mean ± SD values of triple determinations). These results indicate that LPE contains phenolic compounds and flavonoids that may be closely associated with the beneficial actions of* Loranthus parasiticus*.

### 3.2. Inhibitory Effect of LPE on IgE-Mediated Degranulation in RBL-2H3 Cells

**Figure 1:**
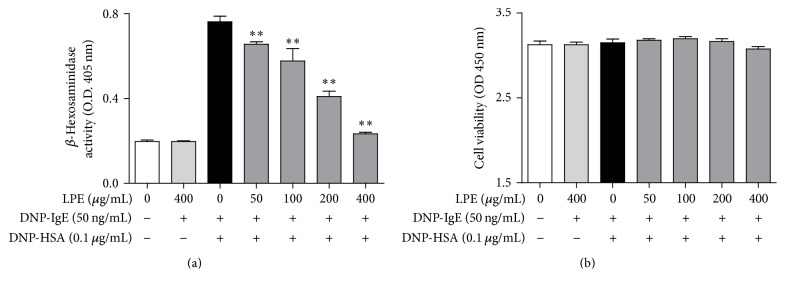
Effect of LPE on degranulation and cell viability in IgE-mediated RBL-2H3 cells. RBL-2H3 cells were seeded on a 24-well plate (1 × 10^5^ cells/well) or a 96-well plate (1 × 10^4^ cells/well) in MEM-*α* with 10% FBS at 37°C overnight and further incubated with DNP-IgE for 24 h. IgE-sensitized cells were preincubated with LPE (0 to 400 *μ*g/mL) for 1 h and then stimulated with DNP-HSA (0.1 *μ*g/mL) for 4 h. *β*-Hexosaminidase activity and cell viability were determined as described in Section 2. Data are the mean ± SD values of triple or octuple determinations. ^*∗∗*^ *P* < 0.01 versus DNP-HSA-treated group. (a) *β*-Hexosaminidase; (b) cell viability.

Because we found that LPE included total phenolic compounds and flavonoids, we investigated whether LPE can inhibit degranulation of IgE-activated mast cells. When IgE-sensitized RBL-2H3 cells were preincubated with various concentrations of LPE (0 to 400 *μ*g/mL) prior to antigen challenge (0.1 *μ*g/mL DNP-HSA), LPE inhibited the release of *β*-hexosaminidase, a common biomarker of degranulation, in a concentration-dependent manner with an IC_50_ value of 184.5 *μ*g/mL (Figure 1(a)). In addition, 400 *μ*g/mL LPE dramatically suppressed IgE-mediated degranulation to a similar level as the control without significant cytotoxicity (Figure 1(b)). Therefore, these results indicate that LPE possesses antiallergic activity at noncytotoxic concentrations by inhibiting degranulation of IgE-activated mast cells.

### 3.3. Inhibitory Effects of LPE on Production of Pro-Inflammatory Mediators

**Figure 2:**
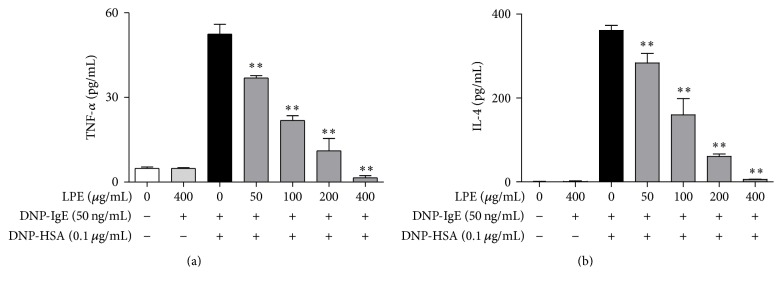
Inhibitory effect of LPE on proinflammatory cytokines. IgE-sensitized RBL-2H3 cells were preincubated with LPE for 1 h prior to antigen challenge. TNF-*α* and IL-4 levels were determined as described in Section 2. Data are mean ± SD values of triple determinations. ^*∗∗*^ *P* < 0.01 versus DNP-HSA-treated group. (a) TNF-*α*; (b) IL-4.

**Figure 3:**
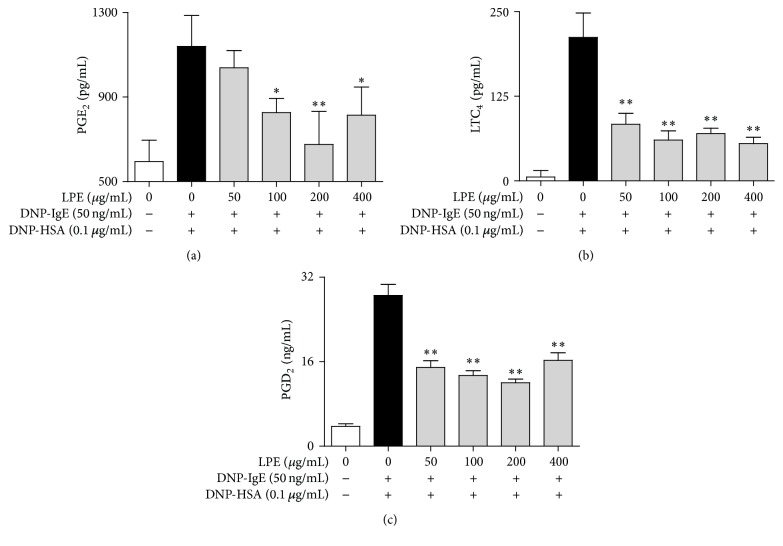
Inhibitory effect of LPE on proinflammatory lipid mediators. IgE-sensitized RBL-2H3 cells were preincubated with LPE for 1 h before antigen treatment. PGE_2_, LTC_4_, and PGD_2_ levels were determined as described in Section 2. Data are mean ± SD values of triple determinations. ^*∗*^ *P* < 0.05 and ^*∗∗*^ *P* < 0.01 versus DNP-HSA-treated group. (a) PGE_2_; (b) LTC_4_; (c) PGD_2_.

Proinflammatory mediators are released from granules in IgE-activated mast cells upon stimulation with antigens [9, 18]. In addition, the mediators are closely associated with the progression of allergic diseases, such as asthma, allergic rhinitis, and atopic dermatitis [8–10, 18]. Therefore, we investigated the effect of LPE on the production of proinflammatory cytokines, such as TNF-*α* and IL-4, and eicosanoids, such as PGE_2_, PGD_2_, and LTC_4_. When IgE-sensitized RBL-2H3 cells were preincubated with LPE before antigen challenge, LPE significantly inhibited the formation of TNF-*α* (IC_50_, 84.27 *μ*g/mL, Figure 2(a)), IL-4 (IC_50_, 93.43 *μ*g/mL, Figure 2(b)), and LTC_4_ (IC_50_, 43.27 *μ*g/mL, Figure 3(b)). In addition, LPE suppressed the biosynthesis of PGE_2_ (IC_50_, 84.10 *μ*g/mL, Figure 3(a)) and PGD_2_ (Figure 3(c)) in a dose-dependent manner up to 200 *μ*g/mL, whereas 400 *μ*g/mL LPE gradually increases the levels of PGE_2_ and PGD_2_. It seems that the effects of LPE at 400 *μ*g/mL may lead to activation of activity or/and expression of PGE_2_ and PGD_2_ synthases. Therefore, further studies are required to develop LPE as a phytomedicine for allergic therapy. Taken together, these findings suggest that LPE inhibits the formation of allergic inflammatory mediators, including proinflammatory cytokines and eicosanoids, but exhibits mild side effects on formation of PGD_2_ and PGE_2_ at a high concentration (400 *μ*g/mL). Consequently, LPE may block acute or chronic inflammation caused by allergic inflammatory mediators in allergic diseases.

### 3.4. Regulatory Effects of LPE on Enzymes for Eicosanoid Biosynthesis

**Figure 4:**
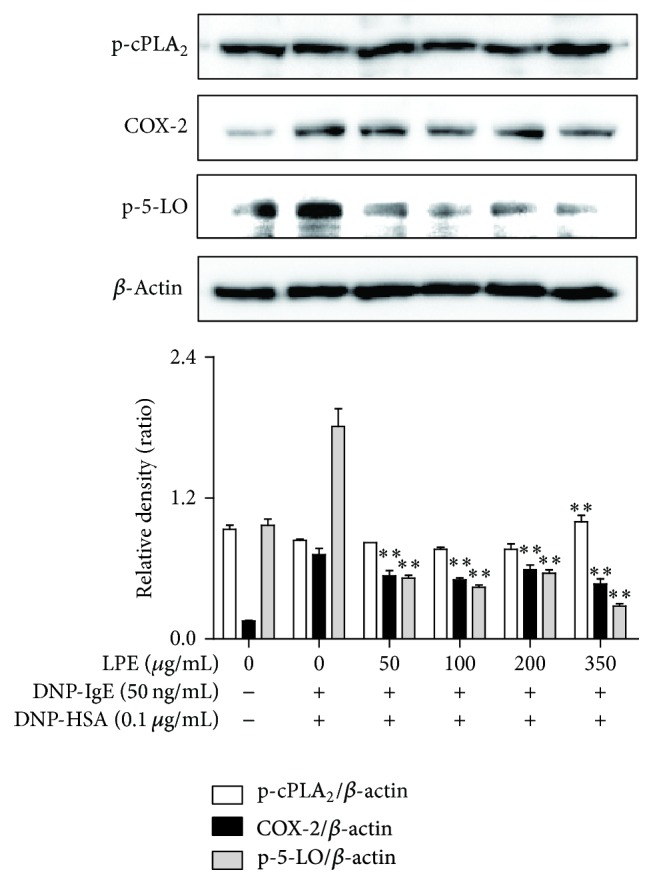
Effect of LPE on phosphorylation or expression of rate-limiting enzymes in the arachidonate cascade. RBL-2H3 cells were seeded on a 6-well plate (5 × 10^5^ cells/well) in MEM-*α* with 10% FBS at 37°C overnight and further incubated with DNP-IgE for 24 h. IgE-sensitized RBL-2H3 cells were preincubated with LPE (0 to 350 *μ*g/mL) prior to antigen challenge. The above cells were washed with 1x DPBS and lysed with cell lysis buffer. The expression of p-cPLA_2_, p-5-LO, COX-2, or *β*-actin was determined as described in Section 2. Similar results were obtained in three independent experiments. ^*∗∗*^ *P* < 0.01 versus DNP-HSA-treated group.

Next, we assessed the effect of LPE on enzymes responsible for biosynthesis of eicosanoids, such as PGE_2_, PGD_2_, and LTC_4_, which induce chronic inflammation in allergic diseases [10, 19, 20]. To address the issue, we examined the effect of LPE on phosphorylation of cPLA_2_, a rate-limiting enzyme of the arachidonate cascade, and 5-LO, a rate-determining enzyme of leukotriene biosynthesis, and the expression of COX-2, a rate-controlling enzyme of prostaglandin biosynthesis. As shown in Figure 4, when IgE-sensitized RBL-2H3 cells were preincubated with various concentrations of LPE for 4 h before antigen exposure, LPE inhibited phosphorylation of 5-LO and expression of COX-2 but not phosphorylation of cPLA_2_. These results indicate that LPE inhibits the biosynthesis of eicosanoids, including PGE_2_, PGD_2_, and LTC_4_, through the regulation of 5-LO and COX-2 activation in prostaglandin and leukotriene biosynthesis, respectively.

### 3.5. Regulatory Effect of LPE on the Activation of the Fc*ε*RI Signaling Cascade

**Figure 5:**
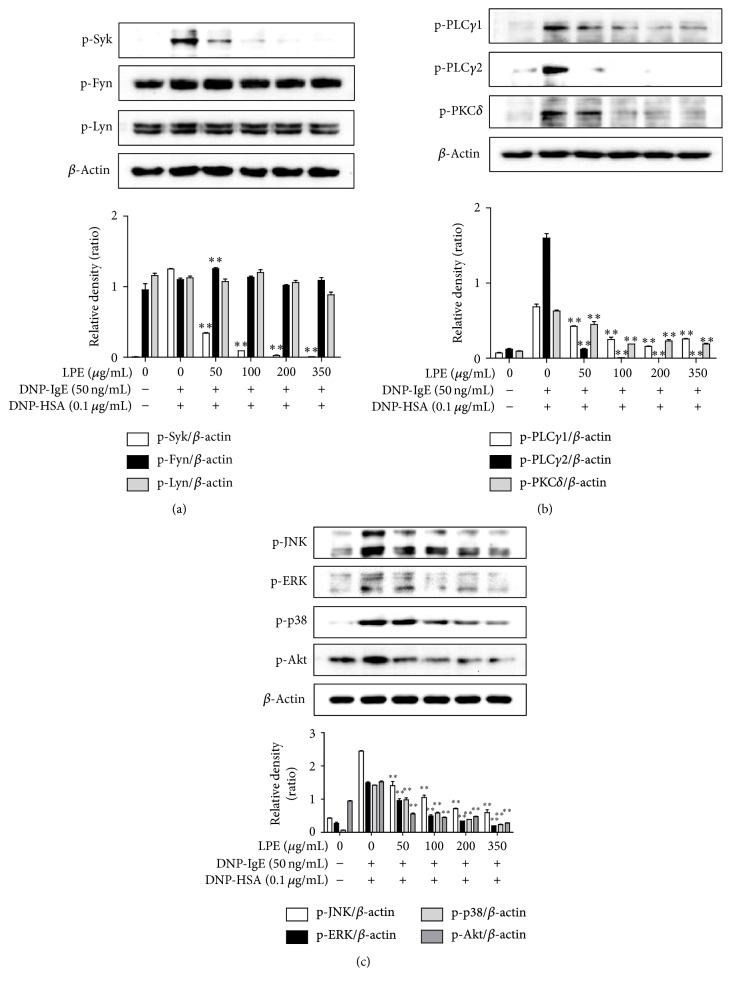
Effect of LPE on phosphorylation of rate-limiting or intermediate proteins in the Fc*ε*RI signaling cascade. IgE-sensitized RBL-2H3 cells were preincubated with LPE for 1 h and then stimulated with antigen for 10 min. The above cells were washed with 1x DPBS and lysed with cell lysis buffer. The expression of p-Lyn, p-Fyn, p-Syk, p-ERK, p-JNK, p-p38, p-Akt, p-PLC*γ*1, p-PLC*γ*2, p-PKC*δ*, or *β*-actin was determined as described in Section 2. Similar results were obtained in three independent experiments. ^*∗∗*^ *P* < 0.01 versus DNP-HSA-treated group. (a) p-Syk, p-Fyn, and p-Lyn; (b) p-PLC*γ*1/2 and p-PKC*δ*; (c) p-JNK, p-ERK, p-p38, and p-Akt.

Finally, because LPE suppressed the rate-limiting enzymes involved in prostaglandin and leukotriene biosynthesis in the late phase (4 h), we further examined the rate-limiting and intermediate proteins related with the Fc*ε*RI signaling cascade in the early phase (10 min) because the activation of eicosanoid biosynthesis is implicated in the Fc*ε*RI signaling cascade in IgE-activated mast cells [17, 21]. As shown in Figure 5(a), when IgE-sensitized RBL-2H3 cells preincubated with LPE were activated by antigen for 10 min, LPE reduced the phosphorylation level of Syk but not Fyn and Lyn, which are initial proteins in the Fc*ε*RI signaling cascade. Furthermore, LPE significantly inhibited the phosphorylation level of PLC*γ*1/2 and PKC*δ*, which are related to the degranulation process (Figure 5(b)), and decreased the phosphorylation levels of ERK, JNK, p38, and Akt, which are related to expression of proinflammatory cytokines (Figure 5(c)). These results suggest that LPE can block activation of the Fc*ε*RI signaling cascade by suppressing the activity of Syk in IgE-activated mast cells.

## 4. Discussion

The action of* Loranthus parasiticus* in allergic reaction is unknown, although it has some beneficial effects [3]. Thus, the present study demonstrates that* Loranthus parasiticus* has antiallergic properties in IgE-activated mast cells based on* in vitro* tests. In addition, such effects of* Loranthus parasiticus* are caused by total phenolic compounds or/and flavonoids, because triterpenes, sesquiterpene lactones, or flavonoids derived from* Loranthus parasiticus* are associated with numerous beneficial effects [3]. Phenolic compounds and flavonoids attenuate allergic responses in IgE-activated mast cells [14, 17]. Nevertheless, the effects of components in* Loranthus parasiticus* on allergic reactions have not been reported.

One possible mechanism for the antiallergic activities of LPE may be related to a direct suppression of activation of the Fc*ε*RI signaling cascade in IgE-activated mast cells because the degranulation initiation of IgE-activated mast cells is closely associated with the activation of the Fc*ε*RI receptor located on the plasma membrane of the cells [7, 8]. Consequently, IgE-activated mast cells liberate various inflammatory mediators, such as IL-4, TNF-*α*, histamine, prostaglandins, and leukotrienes [9, 10, 18, 19]. In support of this, in our study, when IgE-sensitized mast cells were preincubated with LPE prior to antigen challenge, LPE decreased IL-4, TNF-*α*, PGD_2_, PGE_2_, and LTC_4_ production. In addition, LPE inhibited activation of Syk, a rate-limiting intermediate protein of the Fc*ε*RI signaling cascade [8]. Moreover, LPE also suppressed activation of the PLC*γ*1/2-PKC*δ* pathway, which is related to degranulation process [8], and Akt, p38, ERK, and JNK, which are associated with cytokine expression [8], in IgE-activated mast cells. Therefore, the activation of both the PLC*γ*1/2-PKC*δ* pathway and intermediate proteins is directly associated with activation of the Fc*ε*RI signaling cascade. Taken together, the antiallergic action of LPE is closely associated with inhibiting Syk activation in the Fc*ε*RI signaling cascade. Therefore, LPE may directly regulate activation of the Fc*ε*RI signaling cascade through inhibition of Syk activation in IgE-activated mast cells.

Another possible mechanism for the antiallergic activities of LPE may be associated with suppression of arachidonate cascade activation in IgE-activated mast cells because the above cells can produce various proinflammatory lipid mediators, such as LTC_4_, PGD_2_, and PGE_2_ [8–10], and release them from numerous granules [11, 12]. Moreover, these lipid mediators lead to chronic inflammation in allergic diseases, such as asthma and allergic rhinitis [7, 10]. Therefore, the regulation of eicosanoid formation is another important factor for the antiallergic properties of LPE. Consistently, LPE reduced biosynthesis of PGE_2_, PGD_2_, and LTC_4_ and suppressed expression of COX-2, a rate-limiting enzyme for prostaglandin biosynthesis [17], and activation of 5-LO, an initial enzyme for leukotriene biosynthesis [17], in IgE-activated mast cells. These findings suggest that LPE inhibits the formation of eicosanoids through regulation of rate-limiting enzymes, such as COX-2 and 5-LO. In addition, LPE may regulate other enzymes related with eicosanoid biosynthesis with the exception of cPLA_2_. Such effects of LPE may contribute to the enhancement of its antiallergic properties in allergic responses.

## 5. Conclusions

In this study, we revealed, for the first time, a novel role of LPE in IgE-mediated allergic reactions. We found that LPE has antiallergic efficacy in IgE-activated mast cells and contains numerous total phenolic compounds and flavonoids that are potentially responsible for antiallergic actions. The mechanisms of its antiallergic properties include various targets, such as Syk, Akt, ERK, JNK, p38, PLC*γ*1/2, PKC*δ*, 5-LO, and COX-2. LPE can be used to develop a functional food or a phytomedicine for alleviating allergic diseases. Furthermore, it is necessary to identify the active compounds in* Loranthus parasiticus* that possess antiallergic action.

## Abbreviations

5-LO: 5-Lipoxygenase
Akt: Protein kinase B
COX-2: Cyclooxygenase-2
cPLA_2_: Cytosolic phospholipase A_2_
ERK: Extracellular signal-regulated kinase 1/2
Fyn: Feline yes-related protein
HSA: Human serum albumin
IgE: Immunoglobulin E
IL-4: Interleukin-4
JNK: c-Jun* N*-terminal kinase 1/2
LPE: Extract of* Loranthus parasiticus*
LTC_4_: Leukotriene C_4_
Lyn: Src family protein kinase
PGE_2_: Prostaglandin E_2_
PGD_2_: Prostaglandin D_2_
PKC*δ*: Protein kinase C*δ*
PLC*γ*: Phospholipase C*γ*
Syk: Spleen tyrosine kinase
TNF-*α*: Tumor necrosis factor-*α*.
